# Measures of ejection duration and subendocardial viability ratio in normal weight and overweight adolescent children

**DOI:** 10.14814/phy2.14852

**Published:** 2021-05-15

**Authors:** Nicholas D. Tocci, Scott R. Collier, Marco Meucci

**Affiliations:** ^1^ Department of Health and Exercise Science Appalachian State University Boone NC USA

**Keywords:** adolescents, ejection duration, overweight, subendocardial viability ratio

## Abstract

The aim of our study was to determine how being overweight (OW) affects measures of ejection duration (ED), subendocardial viability ratio (SEVR), and central arterial health in a sample of adolescent children. Thirty‐four sex and age‐matched adolescent children (*n* = 34, 17 OW, age = 14 ± 2 years) participated in one laboratory visit. Anthropometric measures, body composition, and cardiovascular measures including resting heart rate, aortic systolic blood pressure (ASBP), carotid‐femoral pulse wave velocity (cf‐PWV), ED (EDms absolute vs. relative ED%), and the SEVR were ascertained. Transfer functions were applied to obtain ASBP. ED was measured as the time from the beginning of the upstroke of the pulse wave and the dicrotic notch, SEVR as the quotient of the diastolic pressure‐time area to the systolic pressure‐time area, and cf‐PWV as the quotient of distance between carotid‐femoral measurement sites and the transit time of the pulse wave. cf‐PWV was significantly higher in OW compared to normal weight participants (5.13 ± 0.85 vs. 4.53 ± 0.46 m/s respectively; *p *= 0.015, *d* = 0.51). OW adolescents also reported significantly higher values for ASBP (103.1 ± 11.8 vs. 95.7 ± 8.2 mmHg respectively; *p* = 0.043, *d* = 0.72) and significantly lower values of SEVR (114.4 ± 25.9% vs. 132.2 ± 22.0% respectively; *p* = 0.038; *d* = 0.33). Overweight adolescents demonstrated higher cf‐PWV, ASBP, and SEVR then normal weight peers.

## Graphical abstract

Employing non‐invasive measures of cardiac function to identify detrimental changes in cardiovascular health shows promising value in pediatric populations. This study investigated how being overweight affects measures of ejection duration, subendocardial viability ratio, and central arterial health in a sample of adolescent children. Overweight adolescents demonstrate higher cf‐PWV, ASBP, and SEVR then normal weight peers.

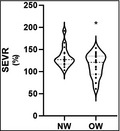

## 1 INTRODUCTION

The rising prevalence of obesity is recognized by the World Health Organization as a major global health issue (Lobstein et al., 2004; Olshansky et al., 2005). Of particular concern is the incidence of obesity in American children which remains a significant public health issue (Hales et al., 2017). Obesity is a well‐established risk factor for the development of cardiovascular disease (CVD) as it increases the risk profile for co‐morbidities including hypertension, type 2 diabetes mellitus, coronary artery disease, and dyslipidemia (Brown et al., 2000; Hubert et al., 1983). Rising evidence suggests that obesity in childhood leads to the development of the same risk factors for disease as adults (Freedman et al., 2007; Tu et al., 2011; Xi et al., 2017).

Compared to normal weight peers, overweight and obese children show elevated systolic (SBP) and diastolic (DBP) blood pressure and as they age are likely to continue a trend of risk of future obesity and hypertension (Brady, 2017; Ewald & Haldeman Ph, 2016; Parker et al., 2016). Children with elevated blood pressure are also more likely to have left ventricular hypertrophy (Brady, 2017), greater arterial stiffening (Tounian et al., 2001) and present with advanced vascular aging (Le et al., 2010). The rising incidence of childhood obesity and hypertension carries the potential to change conventional standards of disease progression, increasing the incidence of CVD earlier in life. An approach recommended by the American Heart Association is the analysis of the central arterial pulse waveforms, and the assessment of pulse wave velocity (PWV), a well‐established clinical measurement of arterial stiffness (Urbina et al., 2009).

Employing non‐invasive measures of cardiac function to identify detrimental changes in cardiovascular health shows promising value in a pediatric population. To achieve the AHA goals, a reliable and non‐invasive assessment of PWV and pulse wave analysis (PWA) is crucial for cardiovascular assessment in a pediatric population (Butlin & Qasem, 2017). Two calculated parameters of PWA identified as strong predictors of CVD in adults but not extensively studied in children are the ejection duration (ED) and the subendocardial viability ratio (SEVR). Historically denoted as left ventricular ejection time, ED is defined as the time in the cardiac cycle during which the left ventricle actively ejects blood through the aortic valve into circulation (Hassan & Turner, 1983). The ED has demonstrated value as a CVD risk assessment in longitudinal studies (Biering‐Sorensen et al., 2018; Haiden et al., 2014) and in the progression of heart failure (Garrard et al., 1970). The SEVR is a clinically useful parameter in adults (Aslanger et al., (2017); Salvi et al., 2013; Scandale et al., 2018; Smith et al., 2012) as it estimates myocardial supply‐demand ratio (Buckberg et al., 1972) and assesses the workload of the heart, which is shown to be higher in overweight and obese individuals (Masuo et al., 2000). Although ED and SEVR are used in adults, previous work on these parameters within pediatric populations is scarce (Marcun‐Varda et al., 2017), and their role as predictor of cardiovascular risk in children and their association with adiposity has yet to be studied extensively.

The purpose of this study is to determine how measures of ED, SEVR, and central arterial health are associated with the overweight condition in a sample of adolescent children.

## 2 MATERIALS AND METHODS

### 2.1 Study population

A sample of healthy boys and girls ages 11–17 years were recruited from the local community. Exclusion criteria were any signs and symptoms of diabetes, heart, respiratory, or renal disease and the use of any medication related to these diseases. Body mass index (BMI) was used as the primary grouping variable.

Sixty‐two adolescent children were selected from the Pediatric Observational Study database. Children with a BMI for age equal to or greater than the 85th percentile were classified as overweight (OW) and children below the 85th percentile were classified as normal weight (NW). The 17 OW participants (11 male, 6 female) in the database were matched with a NW counterpart by sex (first) and by age (second) recorded at the time of testing to maintain samples of comparative size, and to reduce bias during selection. Participants and their parental guardians read and sign a written informed consent to participate in research. This study was approved by the Appalachian State University Institutional Review Board.

### 2.2 Anthropometrics and body composition

Participant's anthropometric and body composition measurements were performed upon arrival at the lab. Body height and weight were measured with a stadiometer to the nearest 0.1 cm and with a digital SECA scale to the nearest 0.1 kg, respectively. BMI was calculated as BMI = kg/m^2^. Body composition was assessed via air displacement plethysmography (BodPod) using the Siri equation and predicted thoracic gas volume. Subjects were instructed to wear tight fitting clothes, wore a rubber swimmers cap, and did not have on shoes or jewelry prior to the measurement. Two measurements of body fat percentage (FM%) were taken, and the average value was used for data analysis.

### 2.3 Measurement of pulse wave analysis and pulse wave velocity

Cardiovascular measurements were obtained from the automated SphygmoCor XCEL (AtCor Medical). Brachial blood pressure measurements and a 10‐s sample of brachial pulse waves were performed after participants laid supine for five minutes in a dimly lit room. Transfer functions were applied to generate a reconstructed aortic pulse waveform (Butlin & Qasem, 2017). All measures of aortic systolic (ASBP), diastolic (ADBP), mean (AMAP), and pulse pressures (APP) as well as resting heart rate (HR), ED and SEVR were derived from the reconstructed aortic waveform. The ED was defined as beginning with the initial upstroke of the forward wave and ending with the occurrence of the dicrotic notch. ED was reported in milliseconds (EDms) and as a percentage of the cardiac cycle (ED%). The Buckberg SEVR was provided by the manufacturer as the quotient of the diastolic pressure‐time area (diastolic area) to the systolic pressure‐time area (systolic area) using the following formula:

$$\text{SEVR}\left(\%\right)=\left(\text{diastolic area}/\text{systolic area}\right)\times 100$$

Carotid‐femoral pulse wave velocity (cf‐PWV) (m/s) was measured in a supine position via applanation tonometry following manufacturer's recommendations. cf‐PWV was calculated by the Sphygmocor XCEL software as the distance between carotid‐femoral measurement sites divided by the transit time of the pulse wave.

### 2.4 Quality control

The SphygmoCor XCEL device provides a quality control index, which represents the reproducibility of the waveforms recorded during PWA. Only recordings including waveforms of consistent amplitude and consistency that resulted in a quality control index equal or greater than 90% were selected and used for the statistical analysis. Measures were performed for each subject a minimum of three times with one‐minute rest intervals and until at least 2 consecutive tests were within ±5 mmHg for brachial systolic and diastolic pressure for PWA (Pickering et al., 2005) and within ±0.3 m/s for cf‐PWV (Butlin & Qasem, 2017). The two closest trials for each participant were averaged and used for the statistical analysis.

### 2.5 Statistical analysis

The dependent variables were analyzed using a one‐way ANOVA by group (NW vs. OW). Assumptions of sphericity were verified using Levene's test for homogeneity of variance. The Cohen's *d* measure of effect size was also determined and reported for all measures of PWA and cf‐PWV. Analyses were performed using SPSS Software (IBM). *A* priori level of significance was set at *p *≤ 0.05. Results are expressed as mean ± SD.

## 3 RESULTS

**FIGURE 1:**
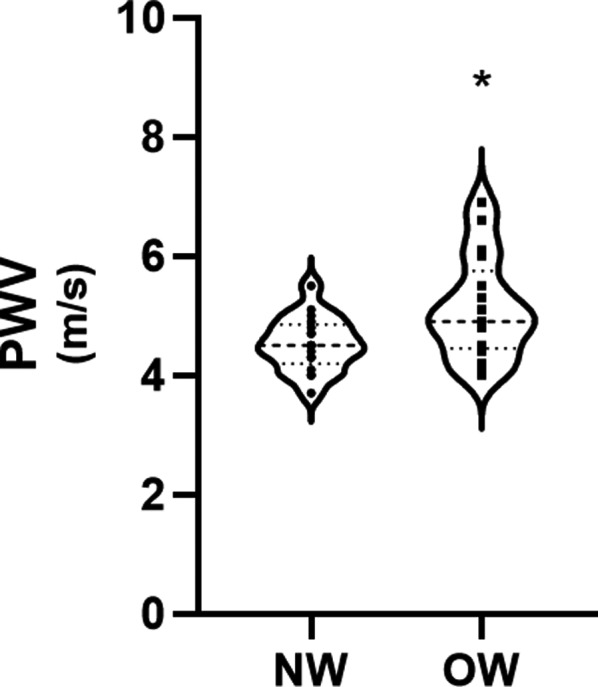
Carotid‐femoral pulse wave velocity (cf‐PWV) in normal weight (NW) and overweight (OW) adolescents. *p < 0.05 compared to normal weight adolescents

**FIGURE 2:**
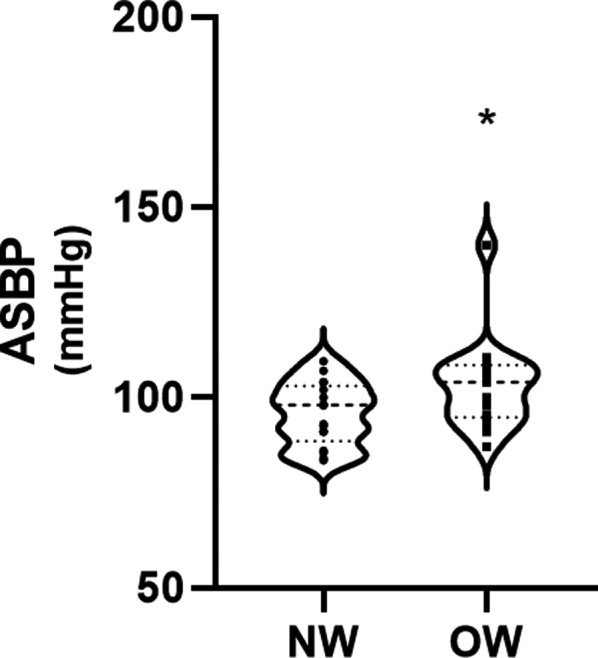
Aortic systolic blood pressure (ASBP) in normal weight (NW) and overweight (OW) adolescents. *p < 0.05 compared to normal weight adolescents

**FIGURE 3:**
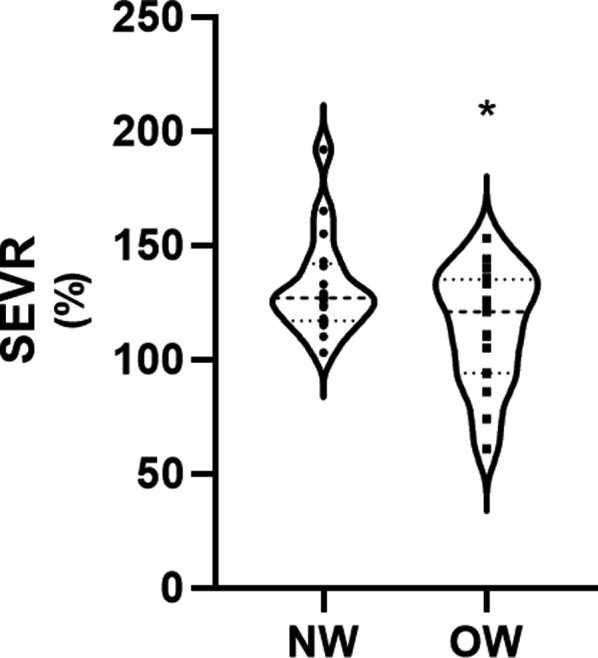
Subendocardial viability ratio (SEVR) in normal weight (NW) and overweight (OW) adolescent. *p < 0.05 compared to normal weight adolescents

Thirty‐four sex and age‐matched participants were included in this study, 17 OW (11 boys and 6 girls) and 17 NW (11 boys and 6 girls) adolescents. The descriptive characteristics of both samples can be seen in Table 1. Cardiovascular measures are reported in Table 2. EDms, ED%, HR, ADBP, AMAP and APP were not significantly different between groups. Compared to the NW group, OW adolescents reported significantly higher measures of cf‐PWV (*p* = 0.015; *F* = 6.6; *d* = 0.88) (Figure 1) and ASBP (*p* = 0.043; *F* = 4.4; *d* = 0.72) (Figure 2) and a significantly lower SEVR (*p* = 0.038; *F* = 4.6; *d* = 0.33) (Figure 3).

**TABLE 1:** Descriptive characteristics and anthropometric measurements of NW and OW participants

	NW (*N* = 17)	OW (*N* = 17)
Age (years)	14.18 ± 2.4	14.08 ± 2.3
Height (cm)	165.3 ± 12.8	166.6 ± 11.7
Weight (kg)	51.8 ± 10.4	82.1 ± 23.7**
BMI (kg/m*^2^*)	18.8 ± 2.2	29.9 ± 5.4**
FM% (%)	17.3 ± 8.7	36.1 ± 10.3**

Data are reported as mean ± SD.

Abbreviations: BMI, body mass index; FM, fat mass; NW, normal weight; OW, over weight.

** *p* < 0.01 compared to normal weight adolescents.

**TABLE 2:** Cardiovascular parameters of NW and OW participants

	NW (*N* = 17)	OW (*N* = 17)	Cohen's *d*	*p*‐value	*F*‐statistic
cf‐PWV (m/s)	4.53 ± 0.46	5.13 ± 0.85	0.88	0.015	6.6
SEVR (%)	132.2 ± 22.0	114.4 ± 25.9	0.33	0.038	4.6
ASBP (mmHg)	95.7 ± 8.2	103.1 ± 11.8	0.72	0.043	4.4
ADBP (mmHg)	63 ± 7	68 ± 10	0.61	0.086	3.1
AMAP (mmHg)	76 ± 7	82 ± 12	0.63	0.075	3.4
APP (mmHg)	33 ± 5	35 ± 6	0.28	0.421	0.7
HR (bpm)	67 ± 9	73 ± 11	0.65	0.066	3.6
ED% (%)	38.8 ± 5.4	41.7 ± 5.9	0.51	0.151	2.2
ED (ms)	346 ± 19	346 ± 19	0.01	0.975	0.0

Data are reported as mean ± SD.

Abbreviations: ADBP, aortic diastolic blood pressure; AMAP, aortic mean arterial pressure; APP, aortic pulse pressure; ASBP, aortic systolic blood pressure; cf‐PWV, carotid‐femoral pulse wave velocity; ED(%), relative ejection duration; ED(ms), absolute ejection duration; HR, resting heart rate; NW, normal weight; OW, over weight; SEVR, subendocardial viability ratio.

## 4 DISCUSSION

The main finding of this study was that overweight adolescents report higher measures of cf‐PWV, ASBP, and SEVR.

We observed higher cf‐PWV and ASBP in OW compared to NW adolescents. The results of our study are in line with the literature indicating that OW children report increased cf‐PWV and ASBP compared to NW children (Celik et al., 2011; Correia‐Costa et al., 2016; Dangardt et al., 2013; Garcia‐Espinosa et al., 2016). Wojtowicz et al. (2017) observed that obese children had higher PWV and central SBP compared to their normal weight counterpart and that the hypertensive children reported the highest aortic SBP, PWV, and left ventricular mass (Wojtowicz et al., 2017). These results suggest the potential for maladaptive cardiac remodeling in these individuals. This change is recognized as unfavorable, as affected adolescents are at higher risk for the development of future CVD (Ayer et al., 2015; Cote et al., 2013).

Our results demonstrate that values of central pressure and stiffness in OW adolescents were accompanied by a significant decrease in the SEVR. To our knowledge, only one pilot study has been conducted to investigate the role of SEVR in the early determination of cardiovascular risks reporting no differences in SEVR between normal weight and overweight children (Marcun‐Varda et al., 2017). Previous research conducted in adults indicate a greater myocardial effort can be observed in obese adults and that the SEVR demonstrates clinical value worth further exploration as an indicator of CVD risk (Smith et al., 2012). Although research does not indicate that SEVR is an indicator of reduced viability of the subendocardium in overweight adolescents, it may be an indicator of greater myocardial effort, especially if low SEVR values occur in conjunction with high ASBP and cf‐PWV. A greater aortic systolic pressure elicits an increase in myocardial work, leading to a decrease in SEVR indicating a potential reduction in myocardial perfusion (Khoshdel & Eshtiaghi, 2019). In a study of hypertensive adults with normal coronary arteries, SEVR was independently related to a reduction in coronary blood flow reserve which was also associated with increased left ventricular mass (Tsiachris et al., 2012). Our results reported no differences in the ED, in milliseconds, between NW and OW participants. These results are unsurprising, since changes in the time of ejection and other systolic time intervals are considerable only in the incidence of severe cardiac remodeling (Weber et al., 2006; Weissler et al., 1968), which is unexpected in our nonclinical population.

Although not statistically significant, OW participants reported higher resting HR compared to the NW group (*p* = 0.066, moderate effect size, *d* = 0.65). Previous research reported that overweight conditions increase metabolic demand (Cote et al., 2013) which is associated with a decrease in vagal tone and an increase in sympathetic activation causing an increase in HR and ASBP (Kalil & Haynes, 2012; Shibao et al., 2007). Moreover, obese children report higher resting HR than normal weight children (Urbina et al., 2009) and BMI has been shown to be directly associated with resting HR in adolescent boys and girls (Baba et al., 2007). In our study, OW participants reported a tendency of lower ED% compared to the NW group (moderate effect size, *d* = 0.51). A lower ED% resulted from an elevated HR in conjunction with preserved EDms which reduced the time spent by the heart in diastole. A reduced diastolic time may contribute to a reduced SEVR which indicates a subtle increase in myocardial stress in our overweight adolescents. Although little is known about the use of SEVR in adolescents, the utility of this measure in predicting the risk of future CVD in overweight children should be further evaluated. Although little is known about the use of SEVR and ED% in overweight adolescents, the utility of combining secondary measures of myocardial effort with primary indicators of cardiovascular health in predicting the risk of future CVD in children should be further evaluated.

The present study has limitations. Our sample is composed of both boys and girls. Moreover, the menstrual cycle in females was not controlled for and it is known to have potential effects on the systolic function (Ounis‐Skali et al., 2006). Given that obesity severity is identified as an important factor in the evaluation of CVD risk (Freedman et al., 2007), future works should emphasize the recruitment of normal weight and obese individuals to study ED and SEVR in pediatric populations. At last, a bigger sample may help identify significant differences in ED%. We presented the F‐statistic to aid the reader in interpreting the significance of the data by our sample size. Measures of PWV and aortic systolic blood pressure (ASBP) combined with SEVR demonstrate utility in the detection of cardiovascular health in adolescents. However, further investigation of these blood pressure waveform derived estimates is warranted to determine the extent of the clinical value of ED and SEVR in relation to ASBP and cf‐PWV in this cohort.

## 5 CONCLUSIONS

This is the first study to investigate blood pressure waveform derived estimates of ED and SEVR in a healthy overweight adolescent population. Overweight adolescents have a lower SEVR compared to normal weight peers accompanied by an increased central arterial stiffness and ASBP. This research provides insight on the utility of these non‐invasive measures for assessing the myocardial supply‐demand ratio in conjunction with arterial stiffness and aortic pressure in overweight adolescents.

## Conflict of interest

The authors declare that there is no conflict of interest.
